# Salmon Migration Patterns Revealed the Temporal and Spatial Fluctuations of the Radiocesium Levels in Terrestrial and Ocean Environments

**DOI:** 10.1371/journal.pone.0100779

**Published:** 2014-06-25

**Authors:** Takaomi Arai

**Affiliations:** Institute of Oceanography and Environment, Universiti Malaysia Terengganu, Kuala Terengganu, Terengganu, Malaysia; Université Pierre et Marie Curie, France

## Abstract

The disabling of the Fukushima Daiichi Nuclear Power Plant (F1NPP) resulted in the release of radionuclides, including ^134^Cs and ^137^Cs, into the air and the ocean. The unpredicted nuclear accident is of global concern for human health and the ecosystem. Although investigations of radionuclides in environments were performed shortly after the accident started, the temporal and spatial impacts and fluctuations on the releasing radionuclides to natural environment remain unclear. I focused on salmon, which migrate from inland to the open ocean globally, to reveal the three-year (May 2011 to February 2014) fluctuations and accumulations of ^134^Cs and ^137^Cs from terrestrial to open ocean environments after the F1NPP accident. The ^134^Cs and ^137^Cs concentrations in six salmonids exhibited lower temporal variations for three years after the F1NPP accident, suggesting that these radionuclides are widely distributed and these radionuclides remain in the natural environment globally with less convergence. The accumulation patterns were significantly different among the different salmon species. Fluvial (freshwater residence) type salmons exhibited significantly higher accumulation in ^134^Cs (25.3–40.2 Bq kg^−1^ in mean) and^ 137^Cs (41.4–51.7 Bq kg^−1^ in mean) than did the anadromous (sea-run) type salmons (0.64–8.03 Bq kg^−1^ in mean ^134^Cs and 0.42–10.2 Bq kg^−1^ in mean ^137^Cs) suggesting widespread contamination in terrestrial environments versus the coastal and open ocean environments. Salmonids are the most highly migratory animals and are characterised by their strong tendency to return home to their natal site for reproduction. Salmonids have a potential to be a good indicator as an effective monitoring animal.

## Introduction

The Great East Japan Earthquake of magnitude 9.0 on Friday 11 March 2011 caused considerable damage in the region, and the large tsunami it created caused much more. Following a major earthquake, a 15-m tsunami disabled the power supply and the cooling of three Fukushima Daiichi reactors, causing a nuclear accident at that date. Radioactive contamination following the Fukushima Daiichi Nuclear Power Plant (F1NPP) accident was the most significant artificial radioactive liquid release into the environment ever known, on a short time and space scale basis [1]–[5]. The amount of radionuclides discharged from the F1NPP immediately after the accident was estimated to be 5–10 PBq to the atmosphere and 3–6 PBq to the sea, the latter being caused by direct leakage of the contaminated cooling water [5]–[8]. Afterwards, trace quantities of radioactive particles from the incident have since been detected around the world, including ^131^iodine and ^134^Cs and ^137^Cs [1], [3]. Fukushima-derived ^134^Cs and ^137^Cs were found throughout a 150,000 km^2^ area of the Pacific Ocean of Japan [4]. In Europe, the first signs of the release were detected seven days later, while the first peak of the activity level was observed between 28 March and 30 March 2011 [3].

Among the radionuclides discharged by the accident, the effects of ^134^Cs and ^137^Cs are the most serious because of their high levels and long decay periods. These radionuclides are considered to most substantially contribute to the contamination of various organisms due to the contamination of their environments. Prior to the accident, the concentrations of ^137^Cs in the surface water of the Pacific Ocean were in the range of 1–4 Bq m^−3^ [9]–[11], which mainly resulted from global fallout due to atmospheric nuclear weapon tests, reprocessing plants, fallout from the accident of the Chernobyl nuclear power plant, and solid waste dumping at sea [12]. Recently, Bailly du Bois et al. [12] proposed that 12–41 PBq of ^137^Cs was discharged to the Pacific Ocean between March 25 and July 28, 2011 and predicted an increase of approximately 6 Bq m^−3^ after dilution in the surface ocean. In addition to this direct release into the ocean, the accident also dispersed approximately 15 PBq of ^137^Cs into the atmosphere, dominated by a leak from 12 March to 6 April 2011 [2]. These nuclides, once discharged into the atmosphere, can have a global effect through fast atmospheric circulation. These phenomena seriously suggest that the contamination by the F1NPP is a global issue and has spread widely throughout the water, soil, air and biota.

Several agencies have conducted investigations to monitor the radioactive quantities of water, soil, air and biota after the F1NPP accident, and considerable data have been reported to understand the present status of radionuclide pollution levels [1]–[4]. Although it has been three years since the disaster by the F1NPP accident, the radionuclide pollution issues have not converged yet, and there is little information about radionuclide behaviour in the natural environment and ecosystem globally. A recent study found that the Pacific blue tuna transported Fukushima-derived radionuclides from Japan to California, and such highly migratory animals transport radionuclides globally as a vector [13], leading to the cause of the distribution of the radionuclides globally. In such a situation, systematic and comprehensive investigations for radionuclides in natural environments are required. However, a number of animals are living in a certain restricted area, e.g., aquatic animals generally can survive in either freshwater or seawater throughout their lives, and thus, it was quite difficult to understand the radionuclide behaviour systematically and comprehensively in various environments from inland water to open ocean environments.

**Figure 1:**
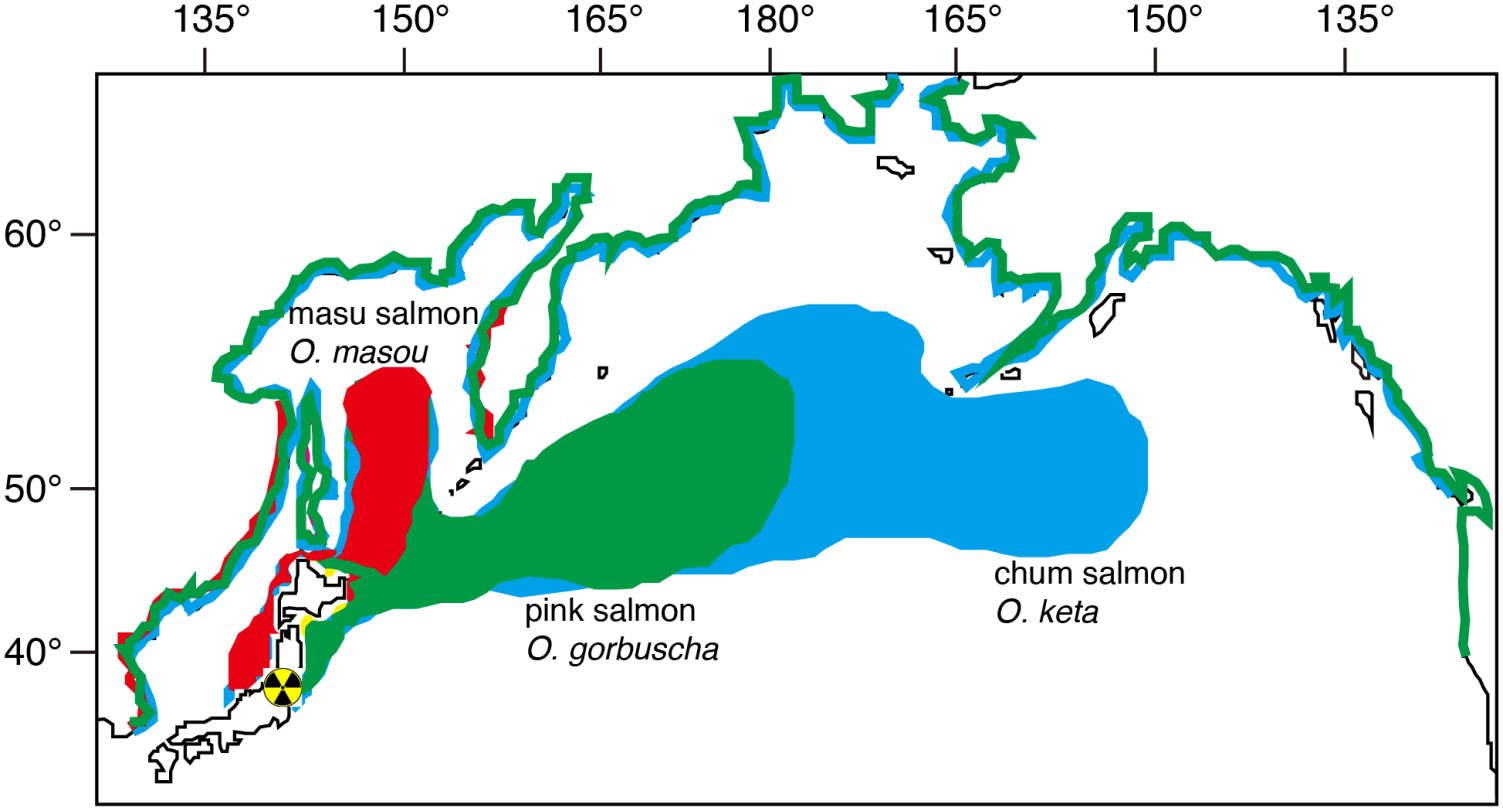
Migratory route and scale from Japanese habitats and the distribution of anadromous salmonids in the Pacific region used in the present study. Chum salmon (blue) have the largest natural range of any Pacific salmon, and undergo the longest migrations within the genus *Oncorhynchus*. Chum are found around the north Pacific, in the waters of Korea, Japan, and the Okhotsk and Bering seas, British Columbia in Canada, and from Alaska to California in the United States. The native range of pink salmon (green) is from the Arctic and Pacific drainages from Mackenzie River delta, the Northwest Territories, to the Sacramento River drainage, California (occasionally as far south as La Jolla, southern California) and in the west from the Lena River in Siberia to Korea. Populations in Asia occur as far south as Honshu in Japan. Masu (cherry) salmon (red) found in the Western Pacific Ocean along East Asia, range from the Kamchatka, Kuril Islands, Sakhalin and Primorsky Krai south through Korea, Taiwan and Japan.

Salmonids are widely distributed in cool and cold waters of all northern continents occurring from the Arctic drainages of Europe, Asia and North America, south as far as the Mediterranean and northern Africa [14]. All of salmonids spawn in freshwater [15] but anadromy (sea-run) is strongly and widely represented. The animal is one of the most studied due to their great importance for food and angling. Thus, the biology and ecology are well understood. Some salmonids highly migrate to far offshore locations, such as the Pacific and Atlantic oceans (Fig. 1); however, all salmons come back to freshwater environments for spawning. Thus, this animal can be useful indicator to monitor for the spread of radionuclides from inland water to open ocean environments without allocation of a significant catching effort and time because a large number of salmons return to their natal rivers.

A number of salmonid species exist in Japan, and the Fisheries Agency of the Japanese Government has examined radionuclides in the animals (Table 1) [16]. In this study, I compared radiocesium (^134^Cs, ^137^Cs and these total) levels among salmonid species using a total of 3,024 samples between May 2011 and February 2014. The levels of accumulation were significantly different in accordance with their life patterns; fluvial (freshwater residence) salmons exhibited the highest level, influenced by the higher radionuclide levels in terrestrial environments, a coastal migrant exhibited a moderate level because the salmon migrate between freshwater and coastal water, and the long-distance migrants to the open ocean exhibited the lowest level, being less impacted by the radiocesium because they live in the open ocean for almost all of their lives after hatching. The present study indicates that migration patterns of the different types of salmon can reveal temporal and spatial fluctuations of radiocesium globally, ranging from terrestrial to open ocean environments.

**Table 1:** Information of life history in the salmonids examined between May 2011 and February 2014 in the present study.

Species	Migration scale(migration type)	Freshwater life periodbefore descending to sea	Marine life periodbefore spawning
*S. leucomaenis*	freshwater	whole life	n/a
(whitespotted charr)	(fluvial)		
*O. masou*	freshwater	whole life	n/a
(masu salmon)	(fluvial)		
*O. nerka*	freshwater	whole life	n/a
(sockeye salmon)	(fluvial)		
*O. masou*	freshwater-coastal	1–2 year(s)	1–2 year(s)
(masu salmon)	(anadromous)		
*O. gorbuscha*	freshwater-coastal-openocean	immediately afterhatching	1–2 year(s)
(pink salmon)	(anadromous)		
*O. keta*	freshwater-coastal-openocean	immediately afterhatching	2–8 years
(chum salmon)	(anadromous)		

n/a: not applicable.

## Materials and Methods

**Figure 2:**
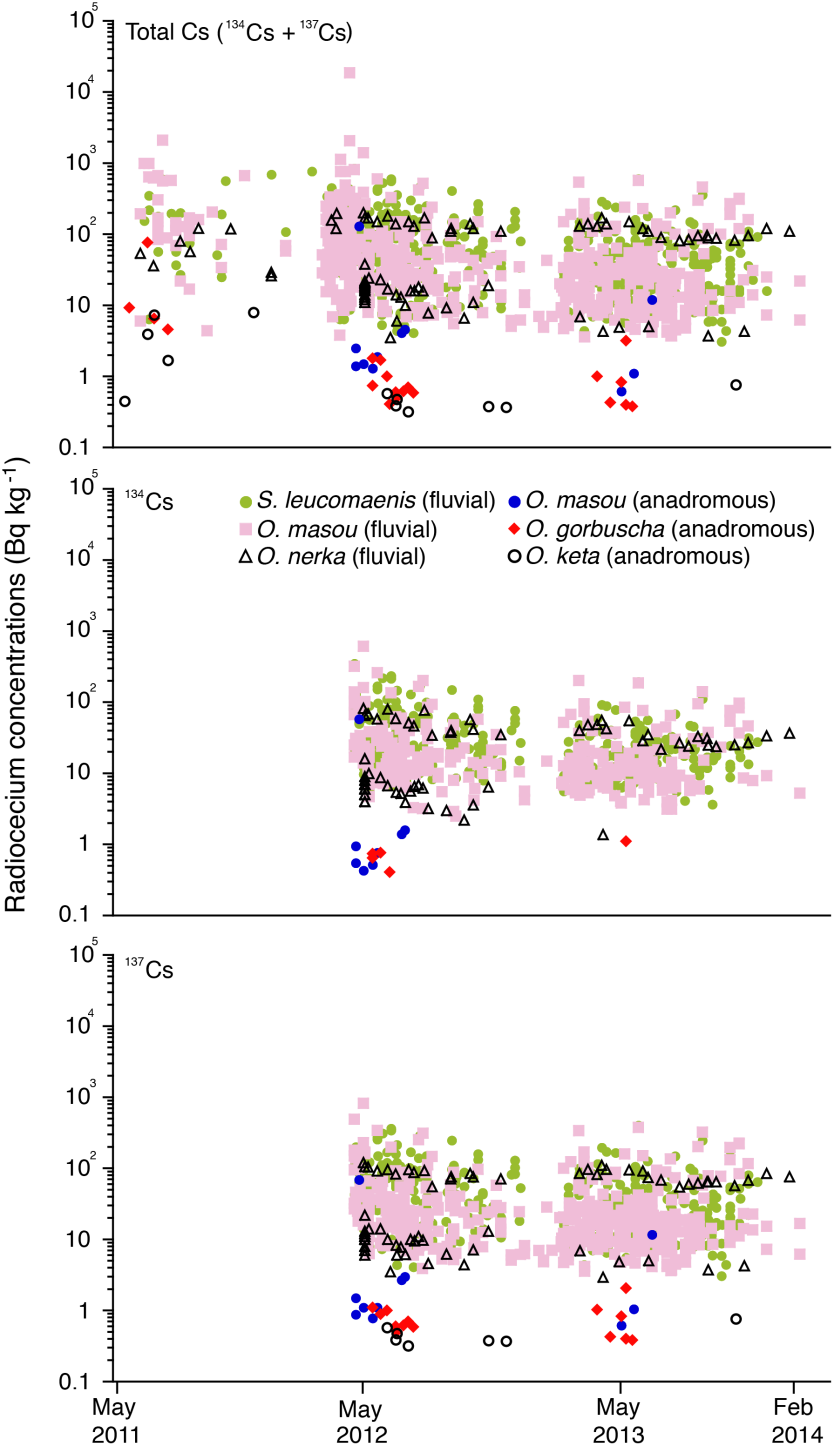
Fluctuations of the radiocesium levels in the total Cs (top) between May 2011 and February 2014, ^134^Cs (middle) and ^137^Cs (bottom) between April 2012 and February 2014 after the F1NPP accident. No significant temporal trends were found for all types of salmon suggesting the continued widespread distribution of these radionuclides with less convergence and indicating that these radionuclides remain in the natural environment for both terrestrial and marine ecosystems. All radiocesium data in the salmonids was from the information published by the Fisheries Agency of the Japanese Government [16] between May 2011 and February 2014. nd: not detectable due to levels below the detection limits.

All radiocesium data in the salmonids was from the information published by the Fisheries Agency of the Japanese Government [16] between May 2011 and February 2014 in the eastern and northern parts of Japan from 3 to 15 prefectures. The radiocesium concentrations were determined as a total between May 2011 and March 2012, while thereafter each ^134^Cs and ^137^Cs concentration was determined in each sample (Fig. 2). All data were shown in the wet weight basis. Each radiocesium concentration among the salmon species was compared using the Kruskal-Wallis test. Significance of the correlation coefficient and regression slope were tested by Fisher’s *Z*-transformation.

## Results

The radiocesium concentrations in six salmonids (five different species and a different migratory type in a same species) (Table 1) exhibited less temporal variations for three (total Cs) or 2 (^134^Cs and ^137^Cs) years after the F1NPP accident (Fig. 2). No significant temporal trends were found for all of the types of salmon (Fisher’s *Z*-transformation, p>0.05), suggesting a continued widespread distribution of these radionuclides with less convergence and that these radionuclides remain in natural environment for both terrestrial and marine ecosystems. Fluvial salmons, such as *Salvelinus leucomaenis*, *Oncorhynchus masou* and *O. nerka*, only reside in a freshwater environment, and thus they accumulate these radionuclides from the terrestrial environment. In contrast, anadromous (sea-run) salmons *O. masou*, *O. gorbuscha* and *O. keta* accumulate radionuclides from terrestrial, coastal and open ocean environments depending on their migration scale (Table 1). The highest concentration of 18,700 Bq/kg in total cesium was found in fluvial *O. masou* in March 2012, one year after the F1NPP accident, and there were still detectable amounts in salmon of over 100 Bq kg^−1^ (Japanese safety limit) of ^134^Cs and ^137^Cs, even three years after the F1NPP incident (Fig. 2).

**Figure 3:**
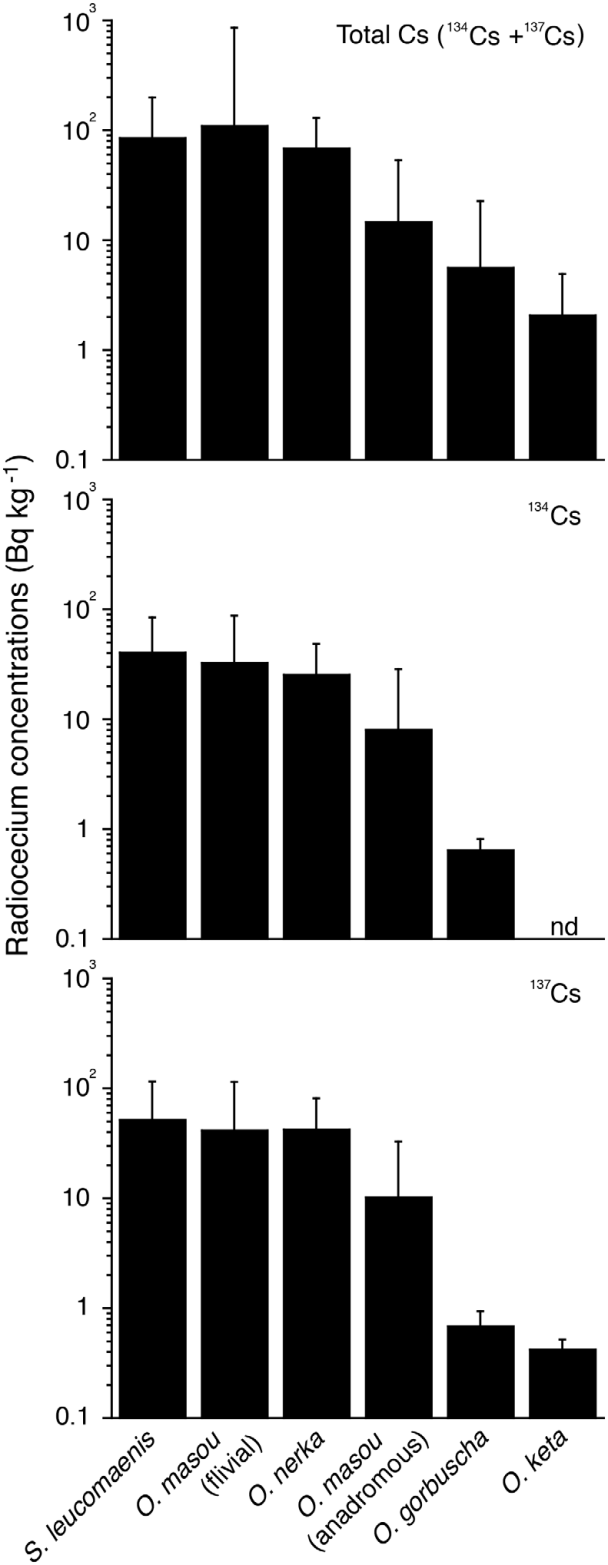
Differences in the radiocesium accumulations among the salmon species between May 2011 and February 2014 after the F1NPP accident. Fluvial salmons exhibited significantly higher concentrations in each radiocesium than those of the anadromous salmon. The detectable rates were also higher in fluvial salmon than in anadromous salmon as presented in Table 2.

However, the accumulation patterns were significantly different among the salmon species. In general, fluvial-type salmon, such as *S. leucomaenis*, *O. masou* and *O. nerka*, exhibited significantly higher accumulation of ^134^Cs (25.3–40.2 Bq kg^−1^ in mean) and^ 137^Cs (41.4–51.7 in mean) than the anadromous-type salmon of *O. masou*, *O. gorbuscha* and *O. nerka* (0.64–8.03 Bq kg^−1^ in mean ^134^Cs and 0.42–10.2 Bq kg^−1^ in mean ^137^Cs) (Table 2, Fig. 3) (p<0.005–0.0001). Among the anadromous salmon, the highest concentration was found in *O. masou*, the second highest was in *O. gorbuscha* and the lowest was in *O. keta* (Table 2, Fig. 3). Furthermore, the detectable numbers were also different among either fluvial- or anadromous- type salmon. There were 529–772 samples of *O. keta* investigated for three years; however, all of the samples were below the detection limit in ^134^Cs, and only seven samples were found to be above the limit. In contrast, radionuclide levels in fluvial salmon could be determined, being generally over 50% (Table 2).

**Table 2:** Radiocesium data information and radiocesium concentrations (above the detection limit) in the salmonids examined between May 2011 and February 2014 in the present study.

Species	Number of the dataabove detection limit(Ratio of detectable (%))			Mean ± SD (Bq/kg)(minimum-maximum(Bq/kg))		
	Total Cs*	134Cs	^137^Cs	Total Cs*	134Cs	^137^Cs
*S. leucomaenis*	524	317	429	84.7±112	40.2±43.5	51.7±62.8
(whitespotted charr)	(70.6)	(45.6)	(61.3)	(3.10–840)	(5.63–350)	(3.07–490)
*O. masou*	670	327	493	109±740	32.4±54.3	41.4±72.2
(masu salmon)	(58.8)	(31.6)	(48.2)	(3.60–18700)	(2.50–610)	(3.57–820)
*O. nerka*	85	68	74	68.4±60.1	25.3±22.7	42.2±38.4
(sockeye salmon)	(88.0)	(73.0)	(82.0)	(3.50–200)	(1.37–82)	(2.94–120)
*O. masou*	11	8	11	14.6±38.4	8.03±20.2	10.2±22.3
(masu salmon)	(11.7)	(10.5)	(18.0)	(0.62–130)	(0.43–58)	(0.62–69)
*O. gorbuscha*	20	5	14	5.60±16.9	0.64±0.16	0.69±0.25
(pink salmon)	(71.6)	(20.2)	(48.4)	(0.38–76.7)	(0.41–1.11)	(0.38–2.07)
*O. keta*	12	0	7	2.06±2.82	nd	0.42±0.09
(chum salmon)	(1.6)	(0)	(1.3)	(0.32–8.00)		(0.32–0.77)

*Total Cs = ^134^Cs+^137^Cs.

Total Cs was available between May 2011 and February 2014.

134 Cs+^137^Cs were available between April 2012 and February 2014.

nd: all samples were below the detection limit.

## Discussion

The present study clearly demonstrated that accumulations of radiocesium were different depending on the migratory patterns and migration scale of the different types of salmon. The highest accumulation was found in the fluvial species (type); their concentrations were similar to those of the other freshwater fish, such as Ayu (*Plecoglossus altivelis*) [17], used to investigate the radiocesium contamination levels in the freshwater ecosystem after the F1NPP accident. Thus, these salmons can also be used to document the ^134^Cs and ^137^Cs contamination levels in the terrestrial environments in East Japan. In contrast to the fluvial salmon, ^134^Cs and ^137^Cs concentrations were quite low in the anadromous salmon, such as *O. masou*, *O. gorbuscha* and *O. nerka.* The detectable ratios of the cesium isotopes were the lowest in *O. keta*, which is the species that has the longest distance of migration, spending almost all of their lives in the North Pacific Ocean (Fig. 1). Among the anadromous salmon, *O. masou* accumulated higher ^134^Cs and ^137^Cs concentrations than those of the other types of salmon. The species remain in freshwater for one to two year(s) after hatching and thereafter go downstream to the sea to growth. In addition, the salmon tends to migrate to a coastal area along the northern Japan for growth (Fig. 1). Thus, anadromous *O. masou* probably have a higher risk for accumulation of radionuclides compared to *O. gorbuscha* and *O. keta. O. gorbuscha* and *O. keta* migrate to sea immediately after hatching, and further migrate along the coastal to the open ocean for growth (Fig. 1); as a result, these salmon probably exhibit a lower risk to accumulate these radionuclides in their lives.

Although the higher levels of radiocesium in fluvial salmons are caused by the serious contamination in the terrestrial environments of East Japan after the F1NPP accident, marine fish are usually approximately 100 times lower in ^137^Cs than are freshwater fish because potassium, which is more abundant in seawater, blocks the uptake of cesium by marine organisms [18]. Thus, the differences in physiological mechanism, especially ionic uptake and elimination of the related osmoregulation, might also accelerate higher ^137^Cs accumulation in the fluvial salmons. The F1NPP accident released large amounts of radioactive substances into the environment and contaminated the soil of Tohoku and the Kanto (East) districts in Japan, and the radiocesium levels in water, soil and biota in terrestrial environment are much more higher than those of the coastal and marine environments [4], [19]. The natural habitats of fluvial salmon, such as *Salvelinus leucomaenis* and *O. masou* are widespread in East Asian countries, as they reside streams, ponds and lakes; as a result, these salmons can be useful indicators for radiocesium pollutions in terrestrial environments.

After the F1NPP accident, the ^137^Cs concentrations in a 30-km perimeter around the plant exceeded 10 Bq L^−1^ [12]. Chum salmon *O. keta* used in the present study might have migrated in the North Pacific at the time of the F1NPP accident because their oceanic migration in North Pacific takes two to eight years. In fact, the *O. keta* ^134^Cs and ^137^Cs concentrations were below the detection limit and 0.42±0.09 Bq kg^−1^ (mean ± SD), respectively, for the 529 samples. These levels were much lower than the highly migratory marine animals of the Pacific blue fin tuna in the North Pacific Ocean (4.0±1.4 Bq kg^−1^ in ^134^Cs and 6.3±1.5 Bq kg^−1^ in ^137^Cs) [13]. The tissue concentrations of radiocesium in the tuna might vary depending on the time spent near the Japanese coast, foraging strategies and the timing of the migration [13]. During open-ocean migration by chum salmon for growth, the salmon has never returned to the Japanese coast and are migrating in the North Pacific Ocean (Fig. 1); and thus the salmon might not accumulate radiocesium of F1NPP accident origin. Nevertheless, trace amounts of radiocesium might have a potential to accumulate during their open ocean migration in the North Pacific Ocean, it could metabolite and eliminate during spawning migration to Japanese coasts. These suggest that the present data of radiocesium levels in chum salmon might be used as a back ground level, and continuous investigations might reveal the fluctuation of radionuclide pollution levels in North Pacific Ocean. The current distribution of chum salmon extends from approximately 45–70°N to 140°E–125°W longitude (Fig. 1). The species has been recorded globally from Russia, United States, Canada, Japan and Korea. Chum salmon has higher homing rate to the natal river, and thus each country could use the salmon to monitor radionuclide levels during their open ocean migration.

The life history, ecology and evolution of salmonid fishes (salmon, trout and char) are dominated by their strong tendency to return home to their natal site for reproduction. Homing characterises the family but is best studied in the anadromous (i.e., ‘sea run’) forms of salmon. Typically, salmon spawn in streams or lakes, and after a variable period of freshwater residence (zero to three years), depending on the species and population, the offspring migrate to the ocean. Salmon remain in the ocean until they begin to mature, and then they return to their natal site to spawn. Thus, salmon can have a great potential to monitor the fluctuation of radionuclides in inland, coastal and open ocean environments as we do not need to catch salmons in coastal and off shore regions because anadromous salmons return to their natal river mouth in spite of their migration scale. Anadromous salmon as well as fluvial salmon are also useful to monitor the radionuclide levels considering the migration scale. In contrast to anadromous salmons, fluvial salmons are suitable indicators for investigating terrestrial environments to reveal the fluctuations of local pollution patterns from the F1NPP event. The present study indicates that the levels of ^134^Cs and ^137^Cs pollution after the F1NPP accident did not converge during the three subsequent years (Fig. 2). Furthermore, Pacific Bluefin tuna, which are highly migratory marine animals, transported Fukushima-derived radionuclides from Japan to California [13], suggesting the widespread distribution of radionuclide pollution globally. Thus, continuous systematic and comprehensive monitoring, taking advantage of the characteristics of salmon migration patterns, might be needed to reveal the temporal and spatial fluctuations of the radionuclides in various environments in the future.

## References

[1] Buesseler K, Aoyama M, Fukasawa M (2011) Impacts of the Fukushima nuclear power plants on marine radioactivity. Environ Sci Technol 45: 9931–9935. 22013920 10.1021/es202816c

[2] Chino M, Nakayama H, Nagai H, Terada H, Katata G (2011) Preliminary estimation of release amount of ^131^I and ^137^Cs accidentally discharged from the Fukushima Daiichi Nuclear Power Plant into the atmosphere. J Nucl Sci Technol. 48: 1129–1134.

[3] Masson O, Baeza A, Bieringer J, Brudecki K, Bucci S, et al (2011) Tracking of airborne radionuclides from the damaged Fukushima Dai-Ichi nuclear reactors by European networks. Environ Sci Technol 45: 7670–7677. 21809844 10.1021/es2017158

[4] Buesseler KO, Jayne SR, Fisher NS, Rypina II, Baumann H, et al (2012) Fukushima-derived radionuclides in the ocean and biota off Japan. Proc Natl Acad Sci USA 109: 5984–5988. 22474387 10.1073/pnas.1120794109 PMC3341070

[5] Estournel C, Bosc E, Bocquet M, Ulses C, Marsaleix P, et al (2012) Assessment of the amount of Cesium-137 released into the Pacific Ocean after the Fukushima accident and analysis of its dispersion in Japanese coastal waters. J Geophys Res 117: C11 doi:10.1029/2012JC007933

[6] Kawamura H, Kobayashi T, Furuno A, In T, Ishikawa Y, et al (2011) Preliminary numerical experiments on oceanic dispersion of ^131^I and ^137^ Cs discharged into the ocean because of the Fukushima Daiichi nuclear power plant disaster. J Nucl Sci Technol 48: 1349–1356.

[7] Miyazawa Y, Masumoto Y, Varlamov SM, Miyama T, Takigawa M, et al (2012) Inverse estimation of source parameters of oceanic radioactivity dispersion models associated with the Fukushima accident. Biogeosci Discuss 9: 13783–13816.

[8] Tsumune D, Tsubono T, Aoyama M, Hirose K (2012) Distribution of oceanic ^137^Cs from the Fukushima Dai-ichi nuclear power plant simulated numerically by a regional ocean model. J Environ Radioact 111: 100–108. 22071362 10.1016/j.jenvrad.2011.10.007

[9] Ikeuchi Y (2003) Temporal variations of ^90^Sr and ^137^Cs concentrations in Japanese coastal surface seawater and sediments from 1974 to 1998. Deep Sea Res 50: 2713–2726.

[10] Nakanishi T, Aono T, Yamada M, Kusakabe M (2010) Temporal and spatial variations of ^137^Cs in the waters off a nuclear fuel reprocessing facility in Rokkasho, Aomori, Japan. J Radioanal Nucl Chem 283: 831–838.

[11] Povinec, Breier R, Coppola L, Groening M, Jeandel C, et al (2004) Spatial distribution of ^3^H, ^90^Sr, ^137^Cs and ^239, 240^Pu in surface waters of the Pacific and Indian Oceans–GLOMARD database. J Environ Radioact 76: 113–137. 15245844 10.1016/j.jenvrad.2004.03.022

[12] Bailly du Bois P, Laguionie P, Boust D, Korsakissok I, Didier D, et al (2012) Estimation of marine source-term following Fukushima Dai-ichi accident. J Environ Radioact 114: 2–9. 22172688 10.1016/j.jenvrad.2011.11.015

[13] Madigan DJ, Baumann Z, Fisher NS (2012) Pacific bluefin tuna transport Fukushima-derived radionuclides from Japan to California. Proc Natl Acad Sci USA 109: 9483–9486. 22645346 10.1073/pnas.1204859109 PMC3386103

[14] McDowall RM (1988) Diadromy in fishes. Croom Helm. 308 p.

[15] Nelson JS (1976) Fishes of the world. Wiley-Interscience. 416 p.

[16] Japanese Ministry of Agriculture, Forestry, and Fisheries (2014) Results of the inspection on radioactivity materials in fisheries products. Available: http://www.jfa.maff.go.jp/e/inspection/index.html. Accessed 2014 Feb 25.

[17] Mizuno T, Kubo H (2013) Overview of active cesium contamination of freshwater fish in Fukushima and Eastern Japan. Sci Rep 3: 1742 doi:10.1038/srep01742 23625055 10.1038/srep01742 PMC3638159

[18] Harte J, Holdren C, Schneider R, Shirley C (1991) Toxics A to Z: A Guide to. Everyday Pollution Hazards. University of California Press. 576 p.

[19] Yasunari TJ, Stohl A, Hayano RS, Burkhart JF, Eckhardt S (2011) Cesium-137 deposition and contamination of Japanese soils due to the Fukushima nuclear accident. Proc Natl Acad Sci USA 108: 19447–19448. 22084074 10.1073/pnas.1112058108 PMC3241755

